# Widespread methanotrophic primary production in lowland chalk rivers

**DOI:** 10.1098/rspb.2013.2854

**Published:** 2014-05-22

**Authors:** Felicity Shelley, Jonathan Grey, Mark Trimmer

**Affiliations:** School of Biological and Chemical Sciences, Queen Mary University of London, Mile End Road, London E1 4NS, UK

**Keywords:** methane oxidation, carbon, photosynthesis, rivers, chemosynthesis

## Abstract

Methane is oversaturated relative to the atmosphere in many rivers, yet its cycling and fate is poorly understood. While photosynthesis is the dominant source of autotrophic carbon to rivers, chemosynthesis and particularly methane oxidation could provide alternative sources of primary production where the riverbed is heavily shaded or at depth beneath the sediment surface. Here, we highlight geographically widespread methanotrophic carbon fixation within the gravel riverbeds of over 30 chalk rivers. In 15 of these, the potential for methane oxidation (methanotrophy) was also compared with photosynthesis. In addition, we performed detailed concurrent measurements of photosynthesis and methanotrophy in one large chalk river over a complete annual cycle, where we found methanotrophy to be active to at least 15 cm into the riverbed and to be strongly substrate limited. The seasonal trend in methanotrophic activity reflected that of the riverine methane concentrations, and thus the highest rates were measured in mid-summer. At the sediment surface, photosynthesis was limited by light for most of the year with heavy shading induced by dense beds of aquatic macrophytes. Across 15 rivers, in late summer, we conservatively calculated that net methanotrophy was equivalent to between 1% and 46% of benthic net photosynthetic production within the gravel riverbed, with a median value of 4%. Hence, riverbed chemosynthesis, coupled to the oxidation of methane, is widespread and significant in English chalk rivers.

## 1. Introduction

Inland waters have received relatively little attention in our attempts to quantify global carbon cycling, compared with the oceanic and terrestrial realms, yet they perform a significant role in carbon sequestration and mineralization [1,2]. Indeed, although modest in their areal extent, the close biogeochemical coupling with terrestrial systems means that globally more carbon is buried in freshwaters than is sequestered on the ocean floor [3]. However, burial is often short-lived as a wide array of microbial communities metabolize the organic carbon and release it back to the atmosphere as either carbon dioxide or methane [4]. Although data for rivers are comparatively scarce compared with lakes [5], many that have been surveyed are often oversaturated in methane and carbon dioxide [6], the partial pressures of which will be influenced by carbon biogeochemistry in the mainstream, groundwater and broader catchment [7,8]. Outgassing of these greenhouse carbon gases from rivers has been widely researched [9,10], but their cycling within rivers and bed sediments has not received as much attention [1].

Traditionally, riverine production is recognized as being supported by a combination of allochthonous carbon from the surrounding catchment and autochthonous carbon produced within the river, both ultimately driven by photosynthesis [11]. Recent work makes the case for a third driver of riverine metabolism, whereby methanotrophy provides a significant portion of carbon to invertebrates in chalk rivers [12], as has been proposed for lakes [13,14]. Such a phenomenon may appear counterintuitive for chalk rivers, being well renowned for their high photosynthetic productivity. Chalk rivers are, however, also oversaturated in methane [15]; the source of methane is thought to be a combination of local methanogenesis in fine sediments [15], and upwelling groundwater which is enriched in methane relative to the atmosphere [16].

Riverbed sediments are known hotspots of biogeochemical cycling, having a concentration of organic matter and micro-organisms several orders of magnitude greater than the overlying water column [17]. Unsurprisingly, then, riverbed epilithic respiration may contribute significantly to whole-stream metabolism [18]. Although a small number of studies have measured dissolved methane in riverbed porewaters [19,20], fewer have measured the potential for methane oxidation within the subsurface gravels. Our previous study at the River Lambourn revealed lower concentrations of methane in the gravel bed porewater than in the main channel, which suggested that the gravel bed is a sink for methane [21]. Thus, in addition to altering the carbon gas balance of emissions from rivers, methanotrophy could account for a significant portion of the primary productivity (i.e. chemosynthetic relative to photosynthetic production). We therefore chose this site to perform a detailed, seasonal study to assess the changing significance of methane-derived carbon as a proportion of photosynthetic production throughout the year. To explore the geographical extent of methane-derived carbon in chalk rivers, we made measurements of methane oxidation and photosynthetic potential in the gravel beds of chalk rivers spanning almost the entirety of the chalk aquifer in southern England.

## 2. Methods

### (a) Study sites and sampling

**Figure 1.**
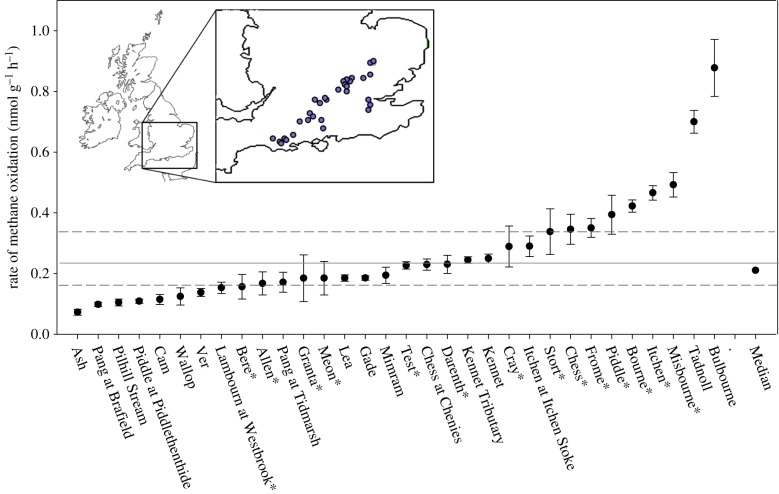
Mean rates of methane oxidation across 32 rivers (±s.e., *n* = 5). The solid line shows the annual average rate from the detailed seasonal study in the River Lambourn, and the dashed lines show the maximum and minimum seasonal rates. Rivers with an asterisk are those for which photosynthetic production was also measured. Inset: the location of these rivers across the chalk aquifer. (Online version in colour.)

Thirty-two chalk rivers with permanent flow, submerged macrophytes and clean gravel beds were selected from across southern England (figure 1). Of these, 15 were chosen for more detailed measurements of benthic photosynthetic and methanotrophic carbon fixation. An additional site on the River Lambourn was further selected for a more detailed seasonal study, which consisted of nine sampling trips between October 2010 and September 2011, and the wider survey was performed in August 2011. One of the sites for the one-off survey was also on the River Lambourn and will be referred to as the Lambourn Westbrook.

### (b) River water methane

Dissolved methane concentration in the river water was quantified by taking water samples (*n* = 5) from the middle of the channel at mid-depth using polytetrafluoroethylene tubing attached to a 60 ml gas-tight syringe. The sample was then immediately discharged into a gas-tight vial (12.5 ml Exetainer, Labco) and allowed to overflow (three times) before being fixed (100 µl ZnCl_2_ 50% w/v; bactericide) and sealed. A 2 ml headspace (analytical-grade helium) was introduced using a two-way valve and gas-tight syringe (Hamilton). After equilibration, gas samples (100 µl) were withdrawn from the headspace and injected into a gas chromatograph fitted with a flame-ionizing detector (Agilent Technologies) [15]. Headspace concentrations of methane were calculated from peak areas calibrated against known standards (Scientific and Technical Gases) and the total amount in the vial (headspace plus water), and thus the river water concentration was calculated using solubility coefficients [22].

### (c) Sediment sampling

To measure potential for methanotrophy, gravels from six discrete locations at each site (*n* = 6) were gently kicked into a fine mesh net, any large stones, detritus and invertebrates were removed, and the sediment was then stored in plastic zip-lock bags and placed into a portable refrigerator for transport back to the laboratory (less than 3 h). At the Lambourn, in order to measure methanotrophy with depth in the riverbed and the quality of allochthonous carbon, sediment cores were taken on each trip using a metal corer (internal dimensions: 18 × 5 cm) manually driven into the riverbed. The sediment core was then extruded and sectioned at 3 cm intervals, the maximum practical spatial resolution. Seven replicate cores (resulting in 35 subsections) were taken on all trips except for October (*n* = 5) and February (*n* = 6). Grain size was determined by sieving samples through nine sieves (0.1–5 mm) and weighing the dried fractions.

### (d) Measuring rates of methane oxidation and estimating net methanotrophy

In the laboratory, sediment (approx. 1 g) and river water (5 ml) were transferred into gas-tight vials (12.5 ml Exetainer, Labco) and sealed. The air headspace was enriched with methane (BOC) by adding 300 µl of 10 000 ppm methane in helium to give a final concentration of 450 nmol l^−1^ in the water [12,22]. The concentration of methane in the headspace of each vial was measured by gas chromatography with flame-ionizing detection (Agilent Technologies) [15], immediately after spiking and then every 24 h for 3–5 days [12]. Between measurements, the vials were incubated on rollers (Denley, Spiramix) in a dark and refrigerated room set to 9°C (±1°C) to mimic average river temperature. Following the final measurement, the samples were dried to a constant weight, and all calculated rates of methanotrophy were normalized for dry mass. Control vials were set up to test for any potential for methane oxidation in the river water, which was always found to be negligible [12].

**Figure 2.**
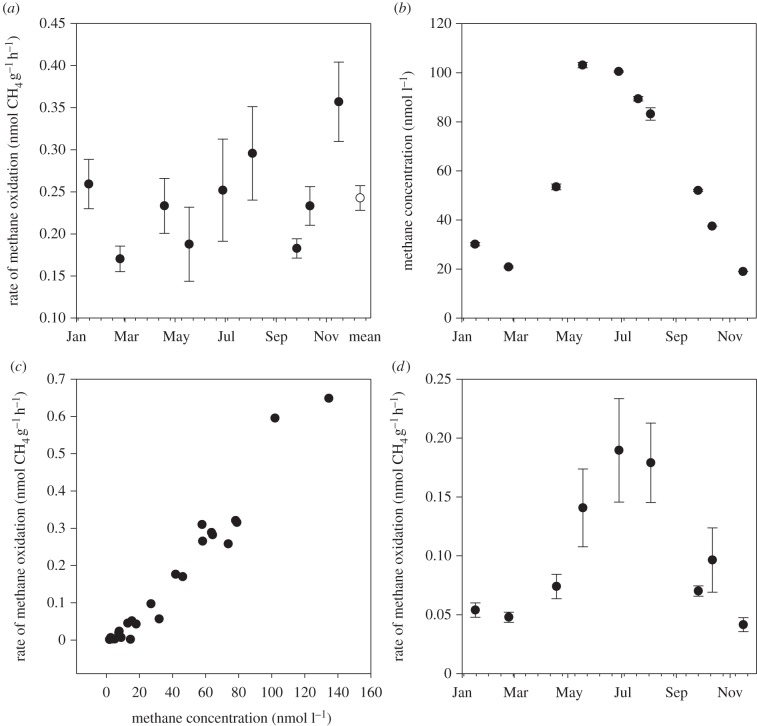
(*a*) Filled circles show mean (±s.e., *n* = 7) rate of methane oxidation across the year under a constant methane concentration, and the open circle is the mean of all data (±s.e., *n* = 60). (*b*) Mean (±s.e., *n* = 5) ambient river water methane concentration. (*c*) Rate of methane oxidation as a function of methane concentration at the start of the incubation. (*d*) Mean (±s.e., *n* = 7) methane oxidation normalized to changing methane concentrations in the river by using the relationship shown in panel (*c*).

The potential for methanotrophy was measured at a constant initial methane concentration in all incubations (across all rivers and throughout the year at the Lambourn). However, the seasonal study showed that the ambient methane concentration in the river displayed strong seasonal variation (figure 2*b*). To investigate the effect of changing methane concentration on methanotrophy, incubations were set up as described above but with varying spikes of methane to give final concentrations in the water ranging from 4 to 80 000 nmol l^−1^. We then used this linear relationship to normalize the measured rates of methane oxidation to the ambient methane concentration for each month. Further, as part of a detailed parallel study using ^13^CH_4_ (Trimmer *et al*. 2013, unpublished results), the carbon fixation efficiency of methanotrophy in these chalk rivers is consistently around 50% (±2%); that is, for each mole of methane oxidized, 50% is fixed as new organic carbon. Accordingly, we multiplied our measured rates of methane oxidation by 0.5 to derive estimates of net methanotrophy to compare with our estimates of net photosynthetic production (NPP; detailed below). Although this is a potential method, performed in the laboratory, the gravels are well irrigated with both methane and oxygen [20], which was captured in our vials, and the strong kinetic effect enabled us to scale the potential activity accordingly. The average rate of methanotrophy for each core (seasonal study, Lambourn) or surface sediment sample (wider survey) was scaled over a depth of 15 cm and surface area of a square metre. We have previously shown that methanotrophy in well-oxygenated riverbeds is not thought to be light-dependent (see §4), unlike stratified water bodies or wetlands where light has indirect effects through changing the position of the oxycline [23], and so hourly rates were multiplied by 24 to scale to daily rates.

### (e) Measuring rates of net photosynthesis

To quantify the potential for photosynthesis in the sediments, we measured oxygen evolution over timed light and dark incubations. Approximately 30 g of each sediment sample was placed inside incubation chambers fitted with a stirrer and a cable gland for holding an oxygen electrode (OX50, Unisense). The chambers were submerged in a temperature-controlled bath (9°C), and the oxygen concentration was logged at 1 min intervals for 45 min in the light (55 µmol quanta m^−2^ s^−1^ at the surface of the gravel), and then the chambers were made dark and logging continued for another 45 minutes (for further details, see [21]). Benthic photosynthetic carbon fixation was calculated by taking one mole of net oxygen production to equate to one mole of carbon fixed. The rates per square metre were multiplied by the average daylight length for the month at the latitude of the study site to give µmol C m^−2^ d^−1^. Given that we could isolate net methanotrophy, we used net photosynthesis to calculate the respective contribution from each to net carbon fixation in the riverbed, as that is what is of greatest significance in terms of export to higher trophic levels.

### (f) Modelling riverbed irradiance and photosynthesis at the River Lambourn

While the laboratory light source remained constant, the light regime at the detailed study site changed seasonally, so we needed to normalize our measured rates of photosynthetic production for *in situ* irradiance by modelling the riverbed light regime using a photosynthesis–irradiance (P–I) curve and riverbed shading data from a previous study [21] (see electronic supplementary material). The ratios between modelled photosynthesis rates for each shading patch type over the annual cycle were used to convert the laboratory data to represent the whole riverbed surface layer instead of just the open gravels. For the August 2011 survey of 15 rivers, we did not produce individual P–I curves for each site, so the estimates of photosynthesis are based solely on laboratory incubations and do not include the effect of shading; hence, we are probably overestimating net benthic photosynthetic production and underestimating the percentage accounted for by net methanotrophy.

With methanotrophic and photosynthetic carbon fixation now in µmol C m^−2^ d^−1^, we divided the former by the latter and multiplied by 100 to give a percentage. When there was no NPP (i.e. respiration outstripped photosynthesis even in the light), methanotrophic C-fixation accounted for 100% of the new carbon produced in the gravels that would still be available to higher trophic levels.

### (g) Quantifying the quality of surface and subsurface chlorophyll *a*

Although light would not penetrate beneath the top 1 cm, and so neither would photosynthetic production, we measured chlorophyll *a* and oxygen evolution at depth (more than 1 cm) to provide a measure of the quality of allochthonous carbon carried into the dark gravel bed. Chlorophyll *a* was extracted three times from the gravels with 30 ml of acetone (90% v/v with ultra-high purity water) over 24 h in a dark refrigerator. Absorbance was measured at 750 nm to check for clarity, and at 650 nm for chlorophyll extinction [24]. We divided the gross oxygen production rates by the chlorophyll *a* content of the gravels to derive biomass-specific photosynthetic production (nmol O_2_ µg^−1^ Chl h^−1^). Here, we used gross photosynthetic production (GPP), because we wanted to quantify the overall capacity of the organisms associated with chlorophyll to produce oxygen.

## 3. Results

### (a) Study site characteristics

At the Lambourn, the temperature of the river water ranged from 6°C in December to 14°C in June, a much smaller range than that of the air temperature, which ranged from −3°C to 28°C, reflecting the strong influence of groundwater typical for these chalk rivers. The macrophytes (predominantly *Ranunculus* spp*.*) and riparian vegetation developed rapidly in late spring and shaded much of the riverbed by June (see electronic supplementary material, figure S1) before dying back in the autumn, as is typical for chalk rivers [25]. There were no seasonal patterns in nutrient concentrations, and the average (*n* = 14) ammonia, nitrate and phosphate concentrations were 2.2 (±0.02 s.e.), 489 (±38 s.e.) and 1.2 (±0.33 s.e.) µmol l^−1^, respectively. Suspended solids remained low throughout the annual cycle (Oct 2010–Sept 2011) at an average of 6 mg l^−1^ (Environment Agency 2013, personal correspondence).

The rivers surveyed in August 2011 covered a wide range of water temperatures (14–20°C), and nitrate (0.2–2 mmol l^−1^), ammonium (3–21 µmol l^−1^) and phosphate (0.2–97 µmol l^−1^) concentrations. The DIC (2.7–4.6 mmol L^−1^) and pH (7.80–8.75) were high across all sites, as would be expected for chalk rivers.

### (b) Dissolved methane concentration and methane oxidation

At all sites, the concentration of dissolved methane in the river water was oversaturated relative to atmospheric equilibration (3.2 nmol l^−1^ at 10°C), ranging from 23 at the Misbourne to 150 nmol l^−1^ at the Piddle. The gravel biofilms oxidized methane at all 32 sites, but the activity varied across rivers, ranging from 0.07 at the Ash to 0.88 nmol CH_4_ g^−1^ h^−1^ at the Bulbourne, both in Hertfordshire (figure 1). The detailed annual study showed that methane concentration was strongly seasonal in the Lambourn, peaking at 103 nmol l^−1^ in late June and falling to 27 nmol l^−1^ in December (figure 2*b*), in agreement with our previous findings [12]. At the Lambourn, the gravels oxidized methane throughout the year (figure 2*a*), but the process was clearly substrate limited, with a linear increase in the rate of methane oxidation both within (figure 2*c*) and well beyond the riverine concentrations (up to 80 µmol CH_4_ l^−1^). This linear relationship was used to normalize the measured rates of methane oxidation at the Lambourn to the methane concentrations measured *in situ* (figure 2*d*). The rates of methane oxidation from the one-off survey in August 2011 were not normalized for ambient methane concentration as the photosynthesis measurements were not be normalized to the ambient light regime. Finally, in the sediment cores from the Lambourn, the rate of methane oxidation decreased significantly with depth into the riverbed (see the electronic supplementary material for table S2), with the rate tending towards zero at 35 cm beneath the surface:3.1
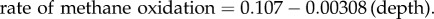
For our calculations on the wider survey, we used the same approach as at the Lambourn seasonal site, integrating over the top 15 cm of the riverbed, as there are few data on subsurface methane and oxygen concentrations in other chalk rivers, or indeed any other river on different geologies.

### (c) Photosynthesis

**Figure 3.**
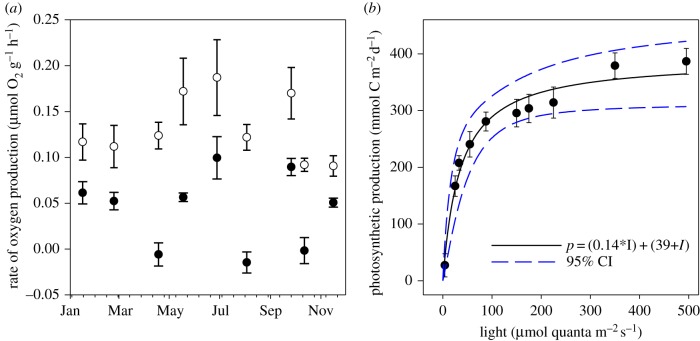
(*a*) Mean (±s.e., *n* = 7) rates of gross (open circles) and net (filled circles) photosynthesis in surface gravels. (*b*) Photosynthesis–irradiance curve for the gravel biofilm community at the River Lambourn (*r*^2^ = 0.92). (Online version in colour.)

Net benthic photosynthetic production was measured in the surface gravels from all 15 of the rivers surveyed in August 2011. Under laboratory conditions, which only simulate completely unshaded parts of the riverbed, the highest production was at the Lambourn at Westbrook (319 nmol O_2_ g^−1^ h^−1^) and the lowest at the Granta (6 nmol O_2_ g^−1^ h^−1^), with the overall range in photosynthetic potential being explained by chlorophyll *a* (i.e. algal biomass). In the Lambourn, gross photosynthesis was measured in the surface sediments throughout the year with the highest rates in summer (figure 3*a*). However, net photosynthesis was observed only in six of the nine months (figure 3*a*). In April, August and October, demand for oxygen via respiration outstripped the production via photosynthesis under illumination, and so the biofilm was net heterotrophic. The P–I curve clearly showed that the biofilm was light saturated at around 100 µmol quanta m^−2^ s^−1^ (figure 3*b*), which means for considerable periods of the summer, the open gravels are fully light saturated. The biomass-specific photosynthetic production (i.e. moles of oxygen produced per unit chlorophyll) remained constant throughout the annual cycle, so we know the photosynthetic kinetics of the biofilm did not vary significantly with season. The modelled benthic photosynthetic activity showed two peaks, one in spring and the other in autumn, with a trough in summer when dense stands of macrophytes heavily shade up to 80% of the riverbed (see electronic supplementary material, figure S2), a pattern widespread across the chalk rivers of southern England.

### (d) Viable subsurface chlorophyll

Chlorophyll *a* was found at all depths within the Lambourn gravels throughout the year, but decreased with depth from 7.4 µg Chl g^−1^ sediment at the surface to 2.8 µg Chl g^−1^ sediment in the deepest section of the cores (table 1). When exposed to light, all subsurface samples were able to produce oxygen, which indicated the presence of viable photoautotrophic organisms at all depths. By normalizing the rate of gross photosynthesis (i.e. taking into account the oxygen consumption via respiration) by chlorophyll content to give biomass-specific photosynthetic production, we found that the quality of the chlorophyll within the riverbed remained constant with depth (table 1). This indicates rapid mixing between the subsurface pore water and overlying surface waters.

**Table 1.** Summary of mean grain size, methane oxidation and chlorophyll quality in the subsurface riverbed of the Lambourn. Here, we have used the biomass specific photosynthetic potential (BSPP) to indicate the viability and quality of chlorophyll delivered to 15 cm into the riverbed. Note the decay in absolute amount of chlorophyll but consistency in BSPP with depth and the slight attenuation in methane oxidation (see §4).

depth interval (cm)	mean grain size (mm)	chlorophyll *a* (µg g^−1^ sediment)	methane oxidation at 450 nM (nmol CH_4_ g^−1^ h^−1^)	gross photosynthetic production (nmol O_2_ g^−1^ h^−1^)	biomass specific photosynthetic production (nmol O_2_ µg^−1^ Chl)
0–3	9.7	7.4	0.723	133	22.5
3–6	6.7	5.6	0.72	79	17.7
6–9	5	3.7	0.576	50	19.9
9–12	4.8	3	0.528	37	20.3
12–15	5.1	2.8	0.507	26	21.5

### (e) Benthic primary production: net photosynthetic versus net methanotrophic carbon fixation

**Figure 4.**
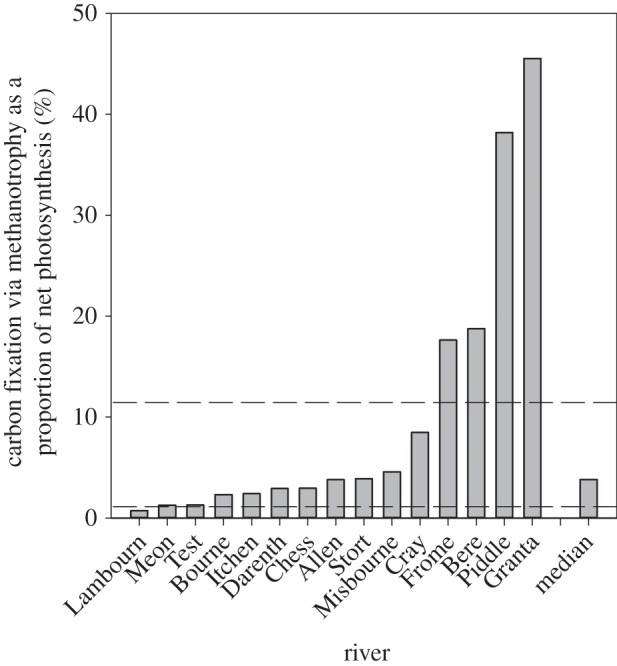
Estimated contribution of methane-derived carbon in the wider survey (assuming 15 cm of methanotrophy). Dashed lines show the maximum and minimum seasonal range of methanotrophic carbon contribution from the detailed seasonal study in the River Lambourn.

Across the 15 rivers, we estimated that between 260 and 960 µmol C m^−2^ d^−1^ was fixed via methane oxidation in August 2011. As a proportion of benthic NPP in the unshaded gravels, net methanotrophy accounted for between 1% and 46% of net carbon fixation (figure 4). This is a conservative estimate as we did not take into account any shading from aquatic macrophytes or riparian vegetation.

**Figure 5.**
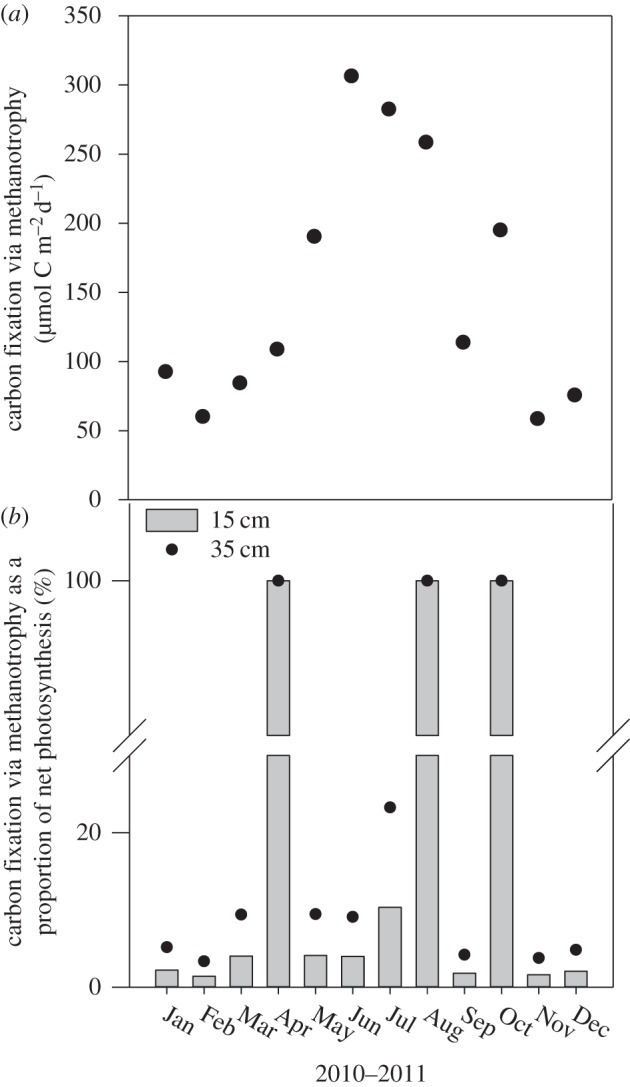
Carbon fixation via methanotrophy in the River Lambourn (*a*) integrated over the top 15 cm of the riverbed, and (*b*) as a proportion of that fixed via photosynthesis both over the first 15 cm (grey bars) and 35 cm (filled circles).

Over the year in the Lambourn, net methanotrophy could potentially fix around 50 and 300 µmol C m^−2^ d^−1^ over the top 15 cm of the riverbed in winter and summer, respectively (figure 5*a*). Once normalized to the ambient methane concentration, the rate of methanotrophic carbon fixation followed the same seasonal pattern as the dissolved methane concentration in the river water, with a peak in summer and a trough in winter. The NPP also peaked in mid-summer, but with no NPP in April, August and October the relationship with season was weaker. As a proportion of carbon fixation via NPP, net methanotrophy fixed between 1% and 11% when there was NPP, and 100% during periods of net heterotrophy (figure 5*b*). This is not to say there was no photosynthesis, but there was no net carbon fixation because of rapid heterotrophic respiration within the biofilm. When integrated over the top 35 cm of riverbed (the inferred extent of methane and oxygen consumption in the riverbed; this study, and see also [20]), the contribution increased by 2.3 times; hence, even when methane concentration in the water was lowest, and thus methanotrophy slowest (February 2011), net methanotrophy could produce the equivalent of greater than 3% of benthic NPP. Annually, carbon fixed via methanotrophy when integrated over the top 35 cm of the riverbed was equivalent to 11% of benthic NPP.

## 4. Discussion

Our study has highlighted geographically widespread methanotrophic carbon fixation within the riverbeds of over 30 chalk rivers. By measuring carbon fixation via photosynthesis, the well-characterized, dominant source of benthic autotrophic carbon fixation in rivers at 15 of these sites, we were able to estimate the contribution of methanotrophy to the production of new biofilm carbon, the grazing community and ultimately the entire ecosystem. Although the input of allochthonous carbon [26], as with most rivers, is an important source of energy to the system, here our focus was the production of new carbon. The decomposition of allochthonous carbon trapped around the macrophyte stands ultimately produces methane [15], which is then available to methanotrophic bacteria as both an energy and carbon source [27]. In this study, we have demonstrated that methanotrophy provides new carbon both at the riverbed surface, where photosynthesis is light limited (especially in summer owing to extensive shading), and deeper down in the riverbed where it is completely dark. Our results indicate a need to re-evaluate the long-held view that rivers receive their carbon through just two major mechanisms: photosynthetic detritus from the catchment (allochthonous carbon) and photosynthetic production within the river itself (autochthonous carbon) [11,28].

While we have shown that the capacity for carbon fixation via methanotrophy in chalk rivers is widespread, it is strongly methane limited, with a linear increase in activity observed well beyond the measured riverine methane concentrations. By contrast, the P–I curve shows that photosynthesis in the open gravels is light saturated for much of the year. In short, in the summer, the photosynthetic organisms cannot exploit the higher light intensities, but the methanotrophs appear to thrive on higher methane concentrations. Photoinhibition studies on methanotrophy have often been in bottle incubations from stratifying water bodies [29,30], where strong gradients of methane and oxygen confound the issue, and high pH (CO_2_ removal owing to high numbers of photosynthetic organisms) in illuminated bottles cannot be ruled out. By contrast, here the riverbed has well-mixed oxygen and methane-rich water, we have previously measured simultaneous photosynthesis and methane oxidation in the laboratory [21], and in our production calculations more than 80% of the length of the sediment core, were from the dark subsurface. Our estimates for photosynthetic production over the 15 riverbeds may be overestimates, because we did not include the effect of shading as we were able to model with greater detail in the Lambourn.

The strong substrate limitation of methanotrophy at riverine methane concentrations implies that the methanotrophs could continue to mitigate the efflux of methane from rivers even where there are hotspots of higher methane concentrations in fine sediment patches [8,15]. Positive correlations between ambient methane concentrations and rates of methanotrophy have also been shown within [31] and among lakes [32], and in wetland sediments [33]. The seasonal pattern in dissolved methane measured here agreed with our previous observations for similar chalk rivers in southern England. Although our seasonal study was restricted to the top 15 cm of the riverbed, data from earlier piezometer work indicated ideal conditions for methanotrophy (i.e. ample oxygen and methane) extend to at least 40 cm into the riverbed [20]; here, we estimate that methanotrophy extends to 35 cm into the riverbed (equation (3.1)), which suggests the data presented in figure 4 are underestimates of the potential contribution of methane-derived carbon to the food webs. The extensive river survey in August covered a greater range of both dissolved methane concentrations and methane oxidation rates, compared with the seasonal range in the Lambourn (figure 1). The methane oxidation rates were all measured with the same starting concentration of methane, and no normalization for ambient methane concentration was carried out on the data; thus, the variation reflects real differences in capacity for methane oxidation across the 32 rivers, and therefore capacity for methanotrophic carbon fixation.

The subsurface measurements of methanotrophy are strong evidence for new carbon fixation at depth and support our previous riverbed porewater gas data, which had suggested a sink for methane at depth in the gravels [12]. We know, however, the dark, subsurface gravels have good hydrological connectivity with the overlying water, as the viability of the chlorophyll pigments measured at depth (table 1) [34] indicates rapid and continual delivery of fresh photoautotrophic organisms. The gravel beds of rivers are known to support a wide array of meiofauna and early ontogenetic stages of macroinvertebrates within the gravel interstices [35], which are likely to graze on both new carbon fixed via methanotrophy and high-quality allochthonous import from above. Given the findings of our study, and by grazing the biofilm at depth, those fauna are likely to play an important role in delivering methane-derived carbon to higher trophic levels.

The seasonal distribution of macrophytes in rivers, and their impact on hydrology and nitrogen cycling, have been studied extensively [36,37], but, as far as we are aware, this is the first study that considers their impact on riverbed primary productivity through shading. The modelled photosynthesis for the whole riverbed shows two peaks, one in spring and the other in early autumn (see electronic supplementary material, figure S2*c*), and is a temporal pattern previously observed for chalk stream secondary production [38]. If the overhanging deciduous vegetation were to be included in the light model, thereby further reducing the summer riverbed irradiances, then the summer trough in photosynthesis would be even deeper and, given the constant yield of oxygen per unit chlorophyll, the mid-summer biofilm could be less photosynthetically productive than those in mid-winter. In short, throughout the annual cycle, both methane oxidation and photosynthesis are limited, by methane concentration and light intensity, respectively.

In combining estimates of both net photosynthetic and methanotrophic production, we placed our measurements of a relatively poorly understood process in the context of the traditionally accepted dominant source of autotrophic carbon fixation in clearwater rivers. At the surface, when the riverbed is illuminated, photosynthetic production completely dominates new carbon fixation. However, no river on Earth has a fully illuminated riverbed, irrespective of hour or season, and thus periods of darkness must be considered. Similarly, in permeable, well-connected and oxygenated riverbeds, one cannot ignore the potential contribution of subsurface carbon fixation (namely via methanotrophy, or even other chemosynthetic metabolism) to the total carbon budget. We have shown that just by considering the top 15 cm of the riverbed at the Lambourn, methanotrophy fixes carbon equivalent to 11% of that fixed via benthic NPP in summer, and conservative estimates from our wider survey suggest elsewhere this rises to at least 46% in August (the highest methane concentrations are usually observed in June). When considering periods of negative NPP, even in the unshaded gravels, we begin to see how important other forms of production may be in these rivers, which are famed for their photosynthetic autochthony.
